# Short- and long-term results of low cost trauma training in a low-income resource-poor country

**DOI:** 10.15694/mep.2018.0000218.1

**Published:** 2018-09-20

**Authors:** Ninos Oussi, Mitra Sadeghi, Javeria S. Qureshi, Charles Mabedi, Peter Elbe, Lars Enochsson

**Affiliations:** 1 Division of Surgery; 2 Department of Vascular Surgery; 3 Department of Surgery; 4 Kamuzu Central Hospital; 5 Department of Surgical and Perioperative Sciences

**Keywords:** Trauma in Africa, Trauma training, Sub-Saharan Africa, Trauma team training

## Abstract

This article was migrated. The article was not marked as recommended.

**Introduction:**Malawi is among the world’s least developed countries. There are 2.1 physicians per 100 000 people and a high trauma-related mortality and morbidity. The lack of healthcare resources requires essential high capacity trauma training at a low cost.

**Methods:**A one-week trauma course was conducted at the Kamuzu Central Hospital in Lilongwe, Malawi. 15 students (13 interns and 2 chief nurses) attended the course. They were trained in initial trauma care, triage and basic practical procedures. Thereafter, evaluated through an identical multiple-choice exam, pre- (PRE) and post-course (POE), following a similar exam 6 months post-course (6MPOE). Prior to, and after the course a confidence-based questionnaire was completed.

**Results:**The participants presented significantly higher test-scores after the course in both POE (26.9±2.6 vs. 22.8±1.6; p=0.0002) and 6MPOE (25.7±2.4 vs. 22.8±1.6; p=0.0028). We also identified the nurses to improve significantly after the course. The highest score of improvement was 27.3%. Higher confidence scores were noticed after the course.

**Conclusion:** This study shows that any healthcare personnel in a low-income setting could benefit from a designed course in trauma management. Thus, we emphasize that healthcare staff undertake similar course to orient towards correct management and assessment of initial trauma patients.

## Introduction

Today there are over 4 million injury-related deaths each year worldwide, where 90% of the injuries occur in low and middle-income countries ( Wesson *et al.*, 2014). Trauma related deaths are almost three times the number of deaths from HIV/AIDS, malaria, and tuberculosis combined ( Kotagal *et al.*, 2014). According to the Global Burden of Disease, injuries cause 11.2% of all disability adjusted life years worldwide ( Kotagal *et al.*, 2014). The incidence of trauma-related morbidity and mortality is increasing and the highest injury trauma-related mortality is seen in sub-Saharan Africa ( MacLeod *et al.*, 2011). Africa has, due to the high volume of surgical disease, the highest percentage per head of surgical disability adjusted life-years (DALYs) in the world ( Lavy *et al.*, 2011).

Depending on the countries economical standard one finds differences in probability of survival. Studies shows that a severely injured patient with mid-range Injury Severity Score has six-times higher risk of mortality in a low-income country, like Ghana, compared to a high-income country, like in Seattle, USA ( Mock, Joshipura and Goosen, 2004).

Malawi is one of the poorest countries in the world with 65% of the population living below 1 US dollar per day (van Amelsfoort *et al.*, 2010) and 93 US dollars in total expenditure per capita on healthcare in 2014 ( World Health Organization, 2018). Like many low-income countries there is paucity of skilled healthcare providers with 2 physicians and 59 nurses per 100 000 people ( World Health Organization, 2008; Qureshi *et al.*, 2013).

Due to the shortage of healthcare workforce in Malawi, surgeons have started to educate clinical officers (non-physician clinicians). To improve clinical care surgeons invested in both regional surgical education but also collaboration with different international partners ( Qureshi *et al.*, 2013).

In Malawi, like in many developing countries about half of the injured patients are within the reproductive ages, in other words; the economically productive segment of the society ( Schultz *et al.*, 2007). Injuries result in a financial burden for the individual as well as the society. However conversely, prior studies have shown young people are most likely to gain full recovery if they receive the correct management and care. Reduction in injury mortality rates in low and middle-income countries to the level of high income countries through better trauma care could save over 2 million lives per year worldwide ( Kotagal *et al.*, 2014). Therefore, the training of hospital staff in trauma management is even more crucial in a setting where there is high trauma-related morbidity/mortality and a resource poor/staff restricted environment. In high-income countries the Advanced Trauma Life Support (ATLS) has been used to standardize and improve trauma care ( Quansah, Abantanga and Donkor, 2008). ATLS, which is managed by the American College of Surgeons, has been spread over more than 80 countries, educating more than 1 million providers so far ( ACS, 2018). The start-up cost for an ATLS course is about 80 000 US dollars, which is not favourable in a poor setting ( Quansah, Abantanga and Donkor, 2008). The ATLS course is also designed with a high technology hospital in mind. In low technology and resource constricted hospitals with limited referral possibilities there is a need for alternative educational approaches ( Nolan, 2005). Several studies of trauma training among healthcare staff in low-income countries have shown positive results ( Schultz *et al.*, 2007; Quansah, Abantanga and Donkor, 2008; Douglas *et al.*, 2010; MacLeod *et al.*, 2011; Kurdin *et al.*, 2018). However, there is a need for follow-up to assess the gained knowledge long term. Here we demonstrate the effectiveness of low cost trauma training to improve healthcare providers trauma management abilities long term.

## Methods

We conducted a one-week initial trauma management and triage course in May 2012 at Kamuzu Central Hospital (KCH), Lilongwe, Malawi. KCH is an 800-bed hospital that serves the central region of Malawi with a population of about 5 million ( Samuel *et al.*, 2010).

Thirteen interns from the surgical department and two Surgery Emergency Room (S-ER) nurses attended the course. The group was homogeneous consisting of 53% female participants and an age ranging from 20-30 years. The inclusion criteria for the participants were all the interns undergoing their surgical rotation at that time due to the current situation in the local setting. At the request of the hospital administration, we also included the two responsible S-ER nurses who served as managers for the emergency room. Before the inclusion they all accepted to take part in the course and the study.

The course was based on ATLS trauma care principles and practical manoeuvres. These covered topics in the standard Airway/neck immobilization (A), Breathing (B), Circulation (C), Disability (D) and Exposure (E) format. The course lectures in ABCDE were held through collaboration with the local staff and expatriate doctors working in Malawi.

Patient triage was held as group seminars with discussion of trauma related cases, where every participant was involved.

We incorporated the theoretical parts with practical procedures and went through fundamental practical procedures such as neck stability, insertion of chest tubes (on goat cadaver), and immobilization of extremities, vascular access and suturing techniques. X-ray interpretations were adapted to the local resources and facilities.

Extra attention was given to paediatric injuries and burn management because of the high amount of paediatric trauma related injuries and burns in the region.

The participants were then evaluated through an identical 33 multiple choice exam pre- (PRE) and post-course (POE) and made a pre/post-course questionnaire for confidence assessment. We were also able to examine them 6 months post-course (6MPOE) with a new 33 multiple-choice exam to evaluate their gained knowledge over time. Unfortunately, we were not able to examine all 15 students as a result of rotations in different district hospitals, insufficient means of communication like no addresses etc., and thus only 11 were examined 6 months post-course (6MPOE).

## Results/Analysis

A total of 15 students, 13 doctors and 2 nurses, returned the multiple-choice exam with 33 questions pre- and post-course but also 6 months post-course. Only 11 doctors participated the 6 months post-course.

The interns had significantly higher test scores after the course in both POE (26.9±2.6 vs. 22.8±1.6; p=0.001) and 6MPOE (25.7±2.4 vs. 22.8±1.6; p=0.003), which is equivalent to previous studies [3]. The improvement range was from 0-27.3% pre- to post-course and 6MPOE from -3 to 21% ( **Table 1**).

**Table 1.** Students (n = 15) exam results; pre-course (PRE), post-course (POE) and six months post-course (6MPOE).

	PRE	POE	6MPOE
1	18	24	
2	23	26	27
3	21	28	21
4	13	19	
5	20	29	25
6	24	25	25
7	24	26	25
8	24	28	
9	24	27	23
10	25	29	29
11	23	27	25
12	24	30	29
13	21	21	26
14	22	24	
15	21	30	28

One of the nurses improved her knowledge with 18% and had similar POE-score as some of the interns.

In similar studies like this the highest gain of scores was in the group with the lowest pre-course results and in our setting this applied to the two nurses.

The confidence score of the participants was increased by 12% after the course. The written course evaluation showed that all were satisfied with the course and recommended that we should include all the interns and even nurses for future courses **( Table 2; Table 3)**.

**Table 2.** Self-assessment Survey (score 1-7).

1.	I’m certain that I can understand the most difficult concepts that are being presented during the course.
2.	I’m certain that I can understand basic terms and facts being brought up during the course (understanding the meaning of the lectures/stations).
3.	I’m certain that I can understand the most complex procedures being taught.
4.	I’m certain that I will succeed at the skills stations.
5.	I’m certain that I will master the skills taught during the course (succeed with the exercise).

**Table 3.** Self-assessment test score: overall test score (n = 15), pre- and post-exam score.

Question	Pre-exam	Post-exam
**1**	5.8	6.8
**2**	6.5	6.8
**3**	5.6	6.6
**4**	6.3	6.7
**5**	6.5	6.8

**Figure 1.**
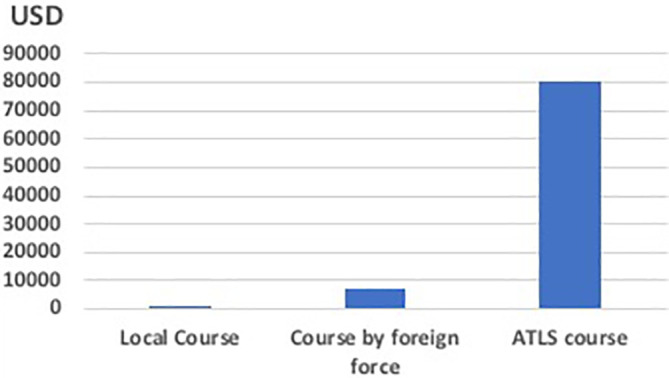
The course expenditures in USD.

The total expenditure on this course was calculated to about 800 US dollars, including material, food and beverage. As the lecturers were mostly local (including the foreign lecturers working at KCH at the moment) there were no expenditures on the salaries. The two Swedish lecturers responsible for the course had salaries from their own employer. This amount is therefore 9 times less the amount of money payed for the two foreign doctors to travel from Sweden to Malawi and arrange the course, and its over 100 times less than setting up an ATLS course **( Figure 1)**.

## Discussion

This study shows that healthcare providers in low-income countries can benefit from trauma training catering to their situation. It is important to include different professional cadres since trauma care is complex and all staff involved should have the same goal in initial management and understand the flow in this chain. In a resource poor setting, staff is required to fill roles they may not fill in a resource rich setting due to the lack of adequate manpower. Interestingly, the two nurses in this study were in the group who gained the most knowledge, showing that staff healthcare providers with less previous trauma experience are more likely to gain the most knowledge. Therefore, it is important to include nurses as well as clinical officers, who serve as non-physician in bigger parts of sub Saharan Africa.

Most importantly, participants retained the knowledge 6 months later. This study was unique in the sense that we could evaluate the participants also after 6 months. This indicates that short-term training can have a long-term effect.

The confidence scores correlate with increased performance of new skills. The participants had high scores in general and showed increased confidence after the course.

Unfortunately, as a cause of economic issues and lack of staff we couldn’t extend the group of students. It’s also difficult for the department to stay without some of their staff for a whole week. For future studies we have to be aware of these types of problems and consider a different strategy to gather a bigger group of staff.

**Limitations:** The limitations of this study are the low number of participants, and also the relatively high number of drop-out rate (27%) due to difficulties reaching them for the 6 months follow up (6MPOE). For future studies it would be more beneficial to include more participants or conduct a multi-centre study.

## Conclusion

This course is similar to an ATLS course covering initial management of trauma patients but in contrast to ATLS it was adjusted to the local settings. By using mainly local doctors instead of international doctors as lecturers the course could be given at a lower cost.

All participants thought the course should include all the interns at KCH as well as be given at an earlier stage of the internship.

The next step is to incorporate more local lecturers into the course and make it part of the intern/nursing curriculum at KCH. It would be most interesting to see if the trauma-care improves over time at KCH as well as at the referring district hospitals by regular trauma training of the interns.

One of the more recent studies in Rwanda ( Petroze *et al.*, 2015) has shown that focused trauma training decreases the mortality rate in injured patients. We need to start placing injury prevention as a major goal for public health management and adjust trauma training to the local settings. There is a need for general improvement in trauma management for all medical staff regardless of degree.

## Take Home Messages

• Healtcare providers in low-income countries can benefit from trauma training without increasing expenses.
• Educating trauma management to the whole team and not only doctors leads to better patient care.
• The participants showed increased confidence after the course.
• Short-term training can have a long-term effect.

## Notes On Contributors

Oussi, Ninos* - MD, PhD-Candidate, General Surgeon and Senior Consultant Surgical Urologist

Sadeghi, Mitra* - MD, PhD-Candidate, General Surgeon, Vascular Surgery Residence

Qureshi, Javeria S - MD, MPH, Assistant Professor, General Surgeon

Mabedi, Charles - MD, Urology Residence

Elbe, Peter - MD, PhD-Candidate, Senior Consultant General Surgeon, Endoscopist

Enochsson, Lars - MD, PhD, Assoc. Professor, Senior Consultant Surgeon, Endoscopist

*Contributed equally to the manuscript.
