# Supplementary material for: Short- and long-term results of low cost trauma training in a low-income resource-poor country
Source: MedEdPublish (2016). 2018 Oct 16;7:218. Originally published 2018 Sep 20. [Version 2] doi: 10.15694/mep.2018.0000218.2 (PMC10704442; doi:10.15694/mep.2018.0000218.2)
Supplement: Peer review history

# Reviewer report: Reviewer response for version 2

**DOI:** 10.21956/mep.20232.r31350

**Published:** 2019-05-08

**Authors:** Trevor Gibbs

**Affiliations:** 1 AMEE

This review has been migrated. The reviewer awarded 4 stars out of 5 Delivering high quality and up-to-date informative courses within difficult circumstances is very difficult and a sensitive issue. I feel that the authors should be congratulated on their efforts here. They have tried to adapt their input to the local needs and also used local resources to keep down costs. I particularly feel that the longer term evaluation shows the true value of the course, even though it was a relatively short course. I would recommend this paper to all concerned with the delivery of such courses in resource constrained countries.

Reviewer Expertise:

NA

No decision status is available

# Reviewer report: Reviewer response for version 2

**DOI:** 10.21956/mep.20232.r31348

**Published:** 2019-03-15

**Authors:** Richard Hays

**Affiliations:** 1 James Cook University

This review has been migrated. The reviewer awarded 4 stars out of 5 This paper reports a nice example of adapting a successful international course to a local situation, using local resources wherever possible to make the course possible. The outcome is good and I particularly like the evaluation after 6 months, which is more meaningful than the more commonly used end-of-course evaluation. Are further evaluation strategies planned, including looking at patient outcomes?

Reviewer Expertise:

NA

No decision status is available

# Reviewer report: Reviewer response for version 2

**DOI:** 10.21956/mep.20232.r31349

**Published:** 2019-03-08

**Authors:** Ken Masters

**Affiliations:** 1 Sultan Qaboos University

This review has been migrated. The reviewer awarded 4 stars out of 5 This is a well written version of the paper, and the author have addressed the previous reviewers’ concerns. The problems of delivering good medical care under these circumstances is well described, and the need for such a course is clearly explained.The standard Pre- and Post-test indicated the short-term impact of the course; I liked the fact that this was followed up with another test 6 months later (the fact that not all candidates could take the later test is unfortunate, but to be expected, especially given the circumstances). Combining a test with the confidence evaluation is particularly useful, as only one or the other would not complete the picture.Figure 1 is probably not entirely necessary, but it does serve to emphasis the massive cost savings for those readers who are unfamiliar with the financial situation of the area.I look forward to seeing papers dealing with further iterations of this course. If the authors do follow up with further courses and papers, it might be useful if they were to supply, as an appendix, a course outline/curriculum.

Reviewer Expertise:

NA

No decision status is available

# Reviewer report: Reviewer response for version 1

**DOI:** 10.21956/mep.19117.r27582

**Published:** 2018-10-04

**Authors:** P Ravi Shankar

**Affiliations:** 1 American International Medical University

This review has been migrated. The reviewer awarded 4 stars out of 5 Thanks for the invitation to review this interesting article. The authors must be commended for their efforts to deliver low-cost trauma training in a resource-constrained setting. I have previously read about clinical officers. The authors can briefly describe the role of these individuals in Malawi’s healthcare system. The authors did not include any clinical officers in their program. What modifications to the ATLS program did the authors carry out which contributed to the substantial reduction in the cost? As the authors correctly point out the sample size was low and the number of nurses was only two. I notice that the study was conducted in 2012. Were there any reasons for this long delay in publishing their findings? I would be interested in knowing if the authors have conducted similar workshops during the period from 2012 to 2018? I and my colleagues had conducted low-cost workshops in Nepal. We were successful in conducting these at a fraction of the cost of official workshops. Language corrections could be carried out in a few places. Educators from developing regions will find this manuscript of special interest.

Reviewer Expertise:

NA

No decision status is available

# Reviewer report: Reviewer response for version 1

**DOI:** 10.21956/mep.19117.r27583

**Published:** 2018-09-21

**Authors:** Bengang Qin

**Affiliations:** 1 The First Affiliated Hospital of Sun Yat-sen University

This review has been migrated. The reviewer awarded 3 stars out of 5 Thank you for inviting me to evaluate the article titled “Short- and long-term results of low-cost trauma training in a low-income resource-poor country”. This is a very interesting and significant paper. Up to now, the population of developing country account for 84 percent of the world population. So, the topic is appropriate to medical education of developing country.1. The abstract is brief and concise description of the information in the manuscript.2. The aim of the paper is clear and practical. the introduction supplies enough background to the paper.3. The methods adequately described to the point. However, it would be better if the paper gives the details and mark the cite of the pre- (PRE) and post-course (POE).4. The result are displayed logically and understandably, however, the author doesn’t mention the statistical method.5. In the first paragraph of the discussion, the sentence “the two nurses in this study were in the group who gained the most knowledge, showing that staff healthcare providers with less previous trauma experience are more likely to gain the most knowledge.” is arbitrary as the samples are not enough. 6. The conclusion is tedious and need to be brief and concise. It recommends placing the paragraph in the conclusion part “The next step is to………” to the discussion part. 7. The following references may be beneficial for the manuscript: (1) Husum H1, Gilbert M, Wisborg T. Training pre-hospital trauma care in low-income countries: the 'Village University' experience. Med Teach. 2003 Mar;25(2):142-8.https://doi.org/10.1080/0142159031000092526(2) Anderson GA, Kayima P, Ilcisin L, Benitez NP, Albutt KH, Briggs SM, Nehra D. Development of a Comprehensive Trauma Training Curriculum for the Resource-Limited Environment. J Surg Educ. 2018, 75(5):1317-1324.https://doi.org/10. 1016/j.jsurg.2018.02.014

Reviewer Expertise:

NA

No decision status is available
